# Phytochemicals and bioactive compounds effective against acute myeloid leukemia: A systematic review

**DOI:** 10.1002/fsn3.3420

**Published:** 2023-05-15

**Authors:** Chukwuebuka Egbuna, Kingsley C. Patrick‐Iwuanyanwu, Eugene N. Onyeike, Johra Khan, Santwana Palai, Sandip B. Patel, Vijaykumar K. Parmar, Garima Kushwaha, Omkar Singh, Jaison Jeevanandam, Suresh Kumarasamy, Chukwuemelie Zedech Uche, Mathiyazhagan Narayanan, Mithun Rudrapal, Uchenna Odoh, Ikenna Chikeokwu, Mihnea‐Alexandru Găman, Kaliyaperumal Saravanan, Jonathan C. Ifemeje, Shahira M. Ezzat, Michael C. Olisah, Chukwudi Jude Chikwendu, Kamoru A. Adedokun, Sikiru O. Imodoye, Ibrahim O. Bello, Hannington Twinomuhwezi, Chinaza Godswill Awuchi

**Affiliations:** ^1^ Africa Centre of Excellence for Public Health and Toxicological Research (ACE‐PUTOR) University of Port Harcourt Port Harcourt Nigeria; ^2^ Department of Biochemistry, Faculty of Science University of Port Harcourt Port Harcourt Nigeria; ^3^ Department of Biochemistry, Faculty of Natural Sciences Chukwuemeka Odumegwu Ojukwu University Anambra Nigeria; ^4^ Department of Medical Laboratory Sciences, College of Applied Medical Sciences Majmaah University Al Majmaah Saudi Arabia; ^5^ Department of Veterinary Pharmacology & Toxicology, College of Veterinary Science and Animal Husbandry OUAT Odisha Bhubaneswar India; ^6^ Department of Pharmacology L.M. College of Pharmacy, Navrangpura Ahmedabad India; ^7^ Department of Pharmaceutical Sciences Sardar Patel University Gujarat Anand India; ^8^ Department of Biotechnology Indian Institute of Technology Roorkee India; ^9^ Department of Chemical Engineering Indian Institute of Technology Madras Chennai India; ^10^ CQM—Centro de Química da Madeira Universidade da Madeira, Campus da Penteada Funchal Portugal; ^11^ PG and Research Centre in Biotechnology, MGR College Tamil Nadu India; ^12^ Department of Medical Biochemistry and Molecular Biology, Faculty of Basic Medical Sciences University of Nigeria Enugu Nsukka Nigeria; ^13^ Division of Research and Innovation Department of Biotecnology, Saveetha School of Engineering SIMATS Tamil Nadu Chennai India; ^14^ Department of Pharmaceutical Sciences, School of Biotechnology and Pharmaceutical Sciences Vignan’s Foundation for Science, Technology & Research Guntur India; ^15^ Department of Pharmacognosy and Environmental Medicines, Faculty of Pharmaceutical Sciences University of Nigeria Nsukka Nigeria; ^16^ Department of Pharmacognosy Enugu State University of Science and Technology (ESUT) Agbani Enugu State Enugu Nigeria; ^17^ Faculty of Medicine "Carol Davila" University of Medicine and Pharmacy Bucharest Romania; ^18^ Department of Hematology Center of Hematology and Bone Marrow Transplantation Bucharest Romania; ^19^ PG and Research Department of Zoology Nehru Memorial College (Autonomous), Puthanampatti (Affiliated to Bharathidasan University) Tamil Nadu Tiruchirappalli India; ^20^ Department of Pharmacognosy, Faculty of Pharmacy Cairo University Cairo Egypt; ^21^ Department of Pharmacognosy, Faculty of Pharmacy October University for Modern Sciences and Arts (MSA) Giza Egypt; ^22^ Department of Medical Biochemistry, Faculty of Basic Medical Sciences Chukwuemeka Odumegwu Ojukwu University, Uli Campus Anambra Nigeria; ^23^ Department of Immunology Roswell Park Comprehensive Cancer Center New York Buffalo USA; ^24^ Department of Oncological Sciences, Huntsman Cancer Institute University of Utah Utah Salt Lake City USA; ^25^ Department of Biological Sciences Southern Illinois University Edwardsville Illinois Edwardsville USA; ^26^ Department of Chemistry Kyambogo University, Kyambogo Kampala Uganda; ^27^ School of Natural and Applied Sciences Kampala International University Kampala Uganda

**Keywords:** acute myeloid leukemia, drug discovery, leukemia, natural products, secondary metabolites

## Abstract

This systematic review identified various bioactive compounds which have the potential to serve as novel drugs or leads against acute myeloid leukemia. Acute myeloid leukemia (AML) is a heterogeneous hematopoietic malignancy that arises from the dysregulation of cell differentiation, proliferation, and cell death. The risk factors associated with the onset of AML include long‐term exposure to radiation and chemicals such as benzene, smoking, genetic disorders, blood disorders, advancement in age, and others. Although novel strategies to manage AML, including a refinement of the conventional chemotherapy regimens, hypomethylating agents, and molecular targeted drugs, have been developed in recent years, resistance and relapse remain the main clinical problems. In this study, three databases, PubMed/MEDLINE, ScienceDirect, and Google Scholar, were systematically searched to identify various bioactive compounds with antileukemic properties. A total of 518 articles were identified, out of which 59 were viewed as eligible for the current report. From the data extracted, over 60 bioactive compounds were identified and divided into five major groups: flavonoids, alkaloids, organosulfur compounds, terpenes, and terpenoids, and other known and emerging bioactive compounds. The mechanism of actions of the analyzed individual bioactive molecules differs remarkably and includes disrupting chromatin structure, upregulating the synthesis of certain DNA repair proteins, inducing cell cycle arrest and apoptosis, and inhibiting/regulating Hsp90 activities, DNA methyltransferase 1, and histone deacetylase 1.

## Graphical abstract

From the data extracted, over 60 bioactive compounds were identified and divided into five major groups: flavonoids, alkaloids, organosulfur compounds, terpenes and terpenoids, and other known and emerging bioactive compounds. The mechanisms of actions of the analyzed individual bioactive molecules differ remarkably and include disrupting chromatin structure, inducing cell cycle arrest and apoptosis, inhibiting Hsp90 activities, DNA methyltransferase 1, and histone deacetylase 1, with the consequent hypomethylation of the DNA.

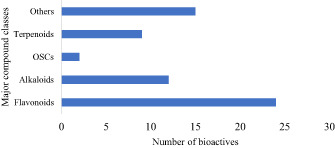

## 1 INTRODUCTION

Leukemia is a malignant proliferation of white blood cells (leukocytes). In acute leukemia, the cells produced by the bone marrow are abnormal, dysfunctional, and fail to mature, passing into the circulation as immature white blood cells called “blasts.” A percentage of ≥20% blasts in the bone marrow is needed to establish the diagnosis of acute leukemia (Chennamadhavuni et al., 2020). Contrastingly, chronic leukemia is characterized by the proliferation of mature, functional leukocytes, and by a small percentage of blasts in the bone marrow (<20%), typically taking months or years. The risk factors for the development of acute leukemia include exposure to ionizing radiation, benzene, chemotherapy (namely topoisomerase inhibitors and alkylating agents), or viral agents (the Epstein‐Barr or the human T‐cell leukemia virus), a previous hematological cancer, or the presence of a genetic syndrome (e.g., Down syndrome). The clinical picture of acute leukemia comprises a conundrum of unspecific symptoms and signs, ranging from fatigue, shortness of breath, fever, recurrent infections, weight loss, bruising, bleeding, and heavy menstrual cycles, to bone pain, hepatomegaly, splenomegaly (or both), and (or) enlarged lymph nodes. To establish a final diagnosis of acute leukemia, the physician often needs to perform a bone marrow biopsy, which guides the patient's further management and the employment of chemotherapy regimens and (or) stem cell transplantation. Several treatment options are reported in Table 1. The prognosis differs based on the type of leukemia studied (Chennamadhavuni et al., 2020).

**TABLE 1:** Selective drugs against acute myeloid leukemia.

S/N	Drug/Agent	Drug type	Mechanism of Action	Monotherapy/ Combination	Ref.
1	Vadastuximab talirine	Monoclonal antibodies	Anti‐CD33 antibody conjugate	Single agent/with standard chemotherapy	Stein et al. (2018)
2	Gemtuzumab ozogamicin	Monoclonal antibodies	Anti‐CD33 antibody conjugate	With standard chemotherapy	Swaminathan and Cortes (2023)
3	CPX‐351	Modification of old drugs	Liposomal formulation of cytarabine and daunorubicin	Single agent	Lancet et al. (2018)
4	Sorafenib	FLT3 inhibitor	Multi‐targeted kinase inhibitor	With standard chemotherapy/with azacitidine	Ohanian et al. (2018)
5	Midostaurin	FLT3 inhibitor	Multi‐targeted kinase inhibitor	With standard chemotherapy	Stone et al. (2017); Weisberg et al. (2020)
6	Lestaurtinib	FLT3 inhibitor	Multi‐targeted kinase inhibitor	With salvage chemotherapy/with standard chemotherapy	Knapper et al. (2017)
7	Quizartinib	FLT3 inhibitor	Second‐generation FLT3 inhibitor	Single agent	Cortes et al. (2018); Georgoulia et al. (2020)
8	Crenolanib	FLT3 inhibitor	Second‐generation FLT3 inhibitor	Single agent/with standard chemotherapy	Galanis et al. (2014)
9	Gilteritinib	FLT3 inhibitor	Second‐generation FLT3 inhibitor	Single agent	Zhao et al. (2019)
10	Ivosidenib	IDH1/2 inhibitor	IDH1 inhibitor	Single agent	Ivosidenib (2018)
11	Enasidenib	IDH1/2 inhibitor	IDH2 inhibitor Adult patients with relapse	Single agent	Stein et al. (2017)
14	Venetoclax	BCL‐2 antagonist	BCL‐2 inhibitor	With low‐dose cytarabine/with HMAs	Bisaillon et al. (2020); Nguyen et al. (2019); Sharon et al. (2019)
15	Guadecitabine	DMNT inhibitor	DNA methyltransferase inhibitor (hypomethylating agent)	Single agent	Kantarjian et al. (2017)
16	Pracinostat	HDAC inhibitor	Histone deacetylase inhibitor	With azacitidine	Novotny‐Diermayr et al. (2012)
17	Aprepitant	NK‐1R antagonist	Induction of cytotoxic and AML cell growth blockade	Single agent	Muñoz and Coveñas (2020)
18	Petromurin C	FLT3 inhibitor	Downregulation of Mcl‐1 in FLT3‐ITD	Single agent	Ha et al. (2020)
19	JSH‐009	CDK9 inhibitor	Anti‐proliferative against AML cell lines	Single agent	Wang, Hu, et al. (2020)
20	LAM‐003	FLT3 inhibitor	Inhibitor against FLT3 inhibitor‐resistant mutants of FLT3	Single agent	Beeharry et al. (2019)
21	Curaxin (CBL0137)	Antagonist toward the toxic effects of MLL/KMT2A gene translocation	Trapping of facilitator of chromatin transcription (FACT) into chromatin, activation of the p53 pathway, and induces an interferon response	Single agent	Somers et al. (2020)

Acute myeloid (or myelogenous) leukemia (AML) is characterized by the abnormal proliferation of blasts of myeloid lineage and has emerged as the most common form of leukemia in the adult population, possessing a bimodal distribution in terms of interested age groups. In 2015, AML affected at least 1 million individuals and caused 147,000 deaths worldwide (Vos et al., 2016). It occurs when a pluripotent hematopoietic stem cell undergoes a malignant transformation and begins to proliferate uncontrollably, giving rise to myeloblasts. Myeloblasts are abnormal white blood cells (Chennamadhavuni et al., 2020), which are immature and poorly differentiated, that are exposed to clonal expansion and proliferation, replacing the normal, healthy cells of the bone marrow. In AML, the bone marrow mostly compromises immature monocytes or granulocytes which in general are positive on the immunohistochemistry tests for markers of myeloid lineage: CD13, CD14, CD15, CD33, CD36, CD61, and CD64 (Chennamadhavuni et al., 2020).

Upon AML diagnosis, other factors are usually considered, such as AML subtypes, age, and the patient's medical history before treatment is scheduled. Usually, the main treatment option for AML is chemotherapy or the use of targeted drugs based on the outcome of medical tests. Some drugs in clinical usage are vadastuximab talirine, gemtuzumab ozogamicin, sorafenib, midostaurin, lestaurtinib, quizartinib, crenolanib, gilteritinib, ivosidenib, enasidenib, venetoclax, guadecitabine, and pracinostat (Beeharry et al., 2019; Bisaillon et al., 2020; Cortes et al., 2018; Galanis et al., 2014; Georgoulia et al., 2020; Ha et al., 2020; Ivosidenib, 2018; Kantarjian et al., 2017; Knapper et al., 2017; Lancet et al., 2018; Muñoz & Coveñas, 2020; Nguyen et al., 2019; Novotny‐Diermayr et al., 2012; Ohanian et al., 2018; Sharon et al., 2019; Somers et al., 2020; Awuchi, 2023;Stein et al., 2017, 2018; Stone et al., 2017; Swaminathan & Cortes, 2023; Wang, Hu, et al., 2020; Weisberg et al., 2020; Zhao et al., 2019; Table 1). Besides drugs, stem cell transplant can also be recommended for treatment. Other conventional forms of cancer treatments such as surgery and radiation therapy are rarely recommended for AML. However, despite the availability of these drugs, the overall survival rate of AML of 5 years is 27.4% according to the National Cancer Institute. This is partly due to drug resistance and increased risk of subsequent cancers and infections. Considering that the current treatment protocols are far from reaching the intended goal, there is an ongoing quest to identify new molecules which could potentially be developed into novel pharmacological agents to battle AML.

Medicinal plants contain chemical compounds called phytochemicals that are good for human health and for the prevention of diseases. The term “phytochemical” is often used to describe chemical substances like antioxidants that may have biological significance but are not recognized as essential nutrients. Some are responsible for color while others are responsible for organoleptic properties in plants. Some phytochemicals have the ability to influence conditions including cancer, stroke, and metabolic syndrome. Thus, this study is aimed at identifying natural bioactive compounds which have been reported to be active against AML.

## 2 MATERIALS AND METHODS

### 2.1 Search strategy

The PubMed/MEDLINE, ScienceDirect, and Google Scholar databases/search engines were searched using the following key terms: (“bioactive compounds” OR “natural compounds” OR “phytochemicals”) AND (“acute myeloid leukemia” OR “AML”).

### 2.2 Inclusion criteria

To be eligible for inclusion, the selected articles had to meet the following inclusion criteria. To begin, the article must have used generally recognized research models (human cell lines and laboratory animals) and reported on the effects of the bioactive compound(s) against AML. Second, the article must have been published in English.

### 2.3 Exclusion criteria

We excluded studies that investigated other types of cancers. Duplicate articles from different databases were screened and only one was retained. Two authors evaluated the title and abstract and references of each article.

## 3 RESULTS AND DISCUSSIONS

### 3.1 Results

**FIGURE 1:**
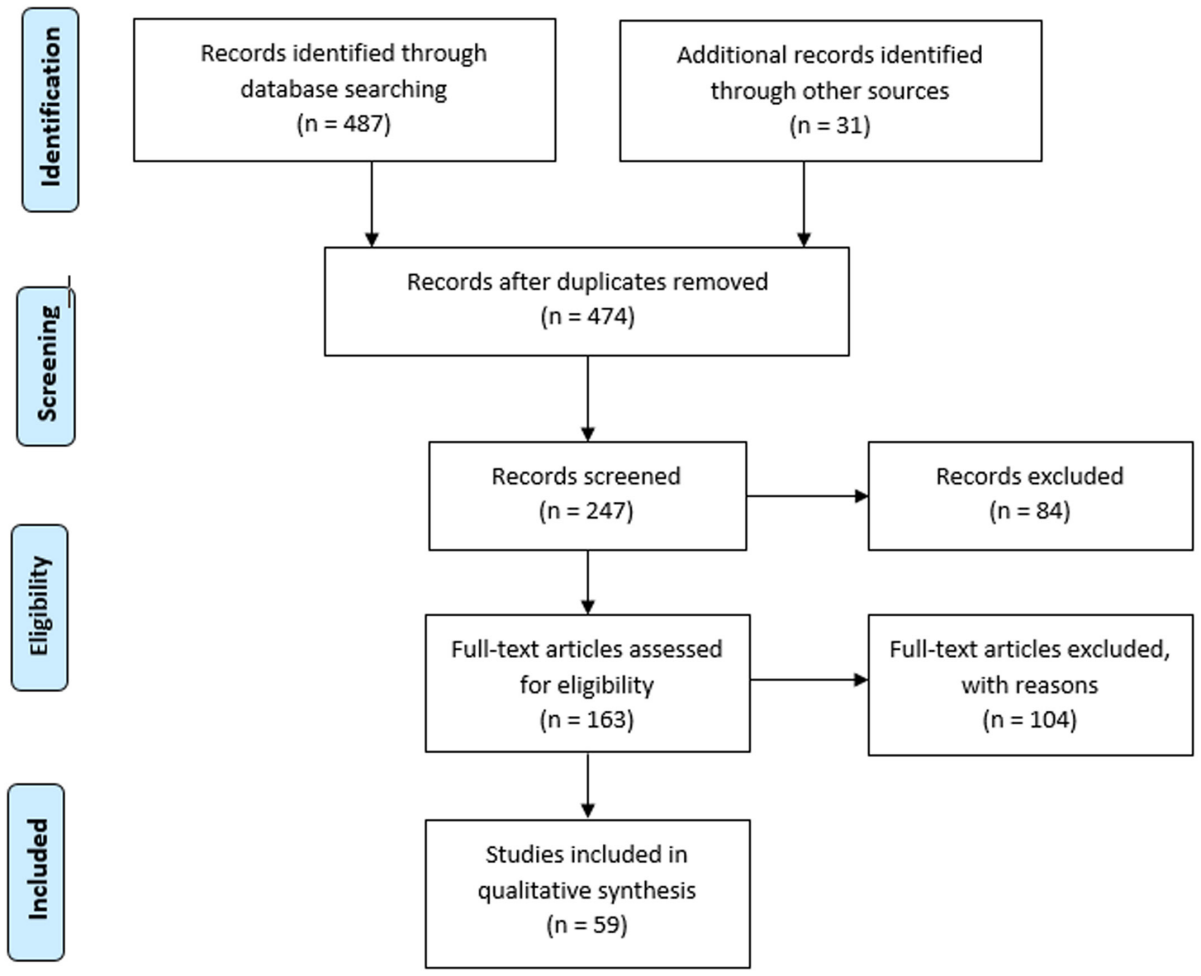
PRISMA flow chart showing the number of selected articles.

**FIGURE 2:**
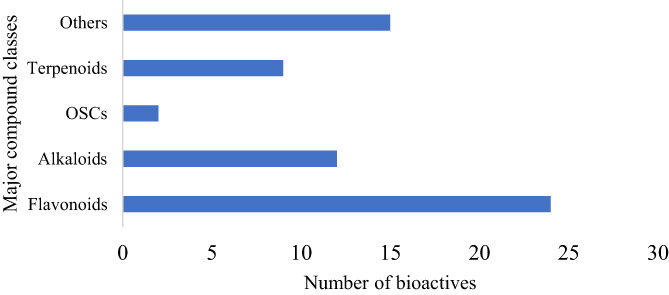
Major compound classes summarized from Table 2.

A total of 518 articles were identified through database searching and other sources. Of this number, 487 papers were identified through database searching while 31 additional articles were identified through other sources (hand searching) (Figure 1). A number of 474 records remained after the exclusion of irrelevant articles and duplicates. After title and abstract screening, a total of 247 articles were left, of which 163 articles were selected after the full‐text assessment. An additional 104 articles were excluded from the study, leaving behind 59 eligible articles for the final analysis. The data obtained from the screening of eligible articles were presented in Table 2. The various bioactive compounds effective against AML, their class, sources, effects, and the nature of the study were presented in this table. According to our analysis, we discovered that the majority of the bioactive molecules fell within the flavonoid class. Overall, five compound classes showed efficacy against AML (Figure 2), namely flavonoids, alkaloids, organosulfur compounds, terpenes, and terpenoids as well as other minor compound classes.

**TABLE 2:** Bioactive compounds active against acute myeloid leukemia.

S/No	Bioactive compound	Class	Sources	Mechanism/effects	Type of study	Research model	References
**Flavonoids**							
1.	Flavokawin B	Chalcone	Kava plant (*Piper methysticum*)	Induced apoptosis in synergy with daunorubicin	In vitro	HL‐60 cell line	Lee et al. (2018)
2.	Hespertin	Trihydroxyflavanone	Oranges, tangerines citrus fruit hybrids, limes, lemons, etc.	Inhibited cell proliferation, induced G2/M arrest, and activated caspase‐3	In vitro	HL‐60 cell line	Adan and Baran (2015)
3.	Fisetin	Flavonol	Strawberries, onions, cucumbers, and apples	Inhibited cell proliferation, G2/M arrest, and caspase 3 activation	In vitro	HL‐60 cell line	Adan and Baran (2015)
4.	Luteolin	Flavone	*Reseda luteola*, thyme, and dandelion	Induced apoptosis	In vitro	HL‐60 and K562 cells	Bangar et al. (2023)
5.	Vitexin	Apigenin flavone glycoside	Chasteberry and pearl millet	Induced apoptosis and upregulated caspase‐3 and − 9 protein expression	In vitro	U937 leukemia cells	Lee et al. (2012)
6.	Wogonin	Flavone	*Scutellaria baicalensis*	Inhibited Cyclin D1, and CDK4	In vitro	U‐937 cells	Zhang et al. (2008)
7.	Chrysin (5,7‐dihydroxyflavone)	Flavone	Honey, propolis, and the passion flowers	Induced apoptosis	In vitro	KG‐1a and K562) cell lines	Mahbub et al. (2013)
8.	Flavopiridol (Alvocidib)	Semisynthetic flavonoid	*Amoora rohituka*,	Decreased peripheral blood blasts	In vivo	Phase II clinical trial	Karp et al. (2007)
9.	Sophoraflavanone G	Tetrahydroxyflavanone	*Sophora flavescens* and *Sophora exigua*	Enhanced apoptosis, including DNA fragmentation and nuclear condensation	In vitro	HL‐60 cell line	Li, Huang, et al. (2016)
10.	Gardenin B derives from a tangeretin	Tetramethoxyflavone	*Gardenia jasminoides Ellis and* tangerines	Activated caspases 3, 8, and 9 and induced apoptosis	In vitro	HL‐60 and U‐937 cells	Cabrera et al. (2016)
11.	Myricetin	Flavonoid	Fruits and vegetables, tea, red wine, nuts, and berries	Promoted Cyt‐C release, induced apoptosis, and decreased the mitochondrial membrane potential	In vitro	HL‐60 cell line	Ko et al. (2005)
12.	Catechin	Flavan‐3‐ol	Cocoa, tea, fruits, prune, and fava bean.	Downregulated the antiapoptotic Bcl‐2 family member Bcl‐xL The pro‐apoptotic member Bax remained unchanged post‐exposure	In vitro	NB4‐R1 and NB4‐R2 cells	Zhang et al. (2014)
13.	Oroxylin A	O‐methylated flavone	*Scutellaria baicalensis* and *Oroxylum indicum*	Upregulated the expression of p21 and C/EBPα Downregulated HDAC‐1 protein levels in vitro and in vivo	In vitro and in vivo	Kasumi‐1 cell line and mice	Hui et al. (2016)
14.	Naringenin	Trihydroxyflavanone	Grapefruit, bergamot, water mint, beans, cocoa, Greek oregano, and tomatoes	Upregulated the expression of p21/WAF1 and induced cycle arrest and apoptosis	In vitro	K562 cells	Li et al. (2015)
15.	Apigenin	Trihydroxyflavone	Parsley, chamomile, and celery	Triggered cell cycle arrest at G2M phase in HL‐60 cells and G0/G1 phase in TF1 cells, induced autophagy in TF1 cells and caspase‐dependent apoptosis in HL‐60 cells, and inhibited the JAK/STAT and PI3K/Akt pathways in both cell lines	In vitro	HL‐60 and TF1	Ruela‐de‐Sousa et al. (2010)
16.	Artocarpesin, cycloartocarpes, and isobavachalcone	Flavonoid	*Artocarpus heterophyllus*	Induced apoptosis through the activation of caspases, and disruption of mitochondrial membrane potential	In vitro	Multiple drug resistance (MDR) cell lines	Kuete et al. (2015)
17.	Quercetin	Flavonoid	Citrus fruits and buckwheat	Inhibited DNMT1 and DNMT3a expression Increased mRNA levels of genes	In vitro and in vivo	HL‐60, U937 cells, and human xenograft AML models	Alvarez et al. (2018)
18.	Morin	Pentahydroxyflavone	Moraceae plants family	Inhibited hepatocytes' transformation by suppressing AP‐1 activity and inducing S‐phase arrest and apoptosis involving a mitochondria‐dependent pathway and a caspase‐3‐mediated mechanism	In vitro	HL‐60 cell	Budisan et al. (2017)
19.	Genistein	Isoflavone	Soybeans and soy products	Arrested mTOR pathway which led to downregulation of protein synthesis.	In vitro	HL‐60 cell	Narasimhan et al. (2015)
20.	Epigallocatechin gallate (EGCG)	Catechin	Green tea	Induced apoptosis	In vitro	HL‐60 and NB4 cells	Britschgi et al. (2010)
21.	Kaempferol	Flavonol	Beans, broccoli, cabbage, grapes, kale, strawberries, and tomatoes	Decreased cell viability and increased subG1 population Decreased expression of Akt, BCL2, ABCB1, and ABCC1 genes, while the expression of CASP3 and BAX/BCL‐2 ratio significantly increased	In vitro	HL‐60 and NB4	Budisan et al. (2017)
22.	Tricetin	Flavone	Pollen of members of the Myrtaceae	Induced apoptosis via ROS‐mediated c‐Jun N‐Terminal kinase activation pathway	In vitro	HL‐60 cell line	Chien et al. (2017)
23.	HLBT‐100 (5,3′‐dihydroxy6,7,8,4′‐tetramethoxyflavanone)	Flavanone	*Tillandsia recurvata* L.	Induced dose‐dependent inhibition activity and apoptosis	In vitro	MV4‐11	Lowe et al. (2017)
24.	Diosmetin	Flavone	Citrus	Induced apoptosis with increase in caspases 8 and 3/7 and the death‐inducing cytokine TNFα	In vitro	OCI‐AML2 and K562	Roma et al. (2018)
**Alkaloids**							
25.	Securinine	Alkaloid	*Securinega suffruticosa*	Induced AML differentiation	In vitro	HL‐60, THP‐1, and OCI‐AML3	Gupta et al. (2011)
26.	Homoharringtonine	Alkaloid	*Cephalotaxus harringtonii*	Induced apoptosis and blocked the cell cycle	In vitro	U937 cells	Tan et al. (2019)
27.	Tetrandrine	Bis‐benzylisoquinoline alkaloid	*Stephania tetandra*	Inhibited MDR1/P‐gp and increased doxorubicin retention	In vitro	NB4 cells	Liu et al. (2015)
28.	Camptothecin	Alkaloid	*Camptotheca acuminata*	Induced c‐FLIPL cleavage and procaspase‐8 activation	In vitro	MV‐4‐11	Bredholt et al. (2009)
29.	Curine	Alkaloid	*Chondrodendron platyphyllum*	Increased the apoptotic cell number. Caused phosphatidylserine externalization and membrane depolarization	In vitro	HL‐60, K562, and HT29 cells	Dantas et al. (2015)
30.	Intermedin A	Alkaloid	*Alpinia intermedia*	Induced apoptosis	In vitro	HL‐60 cell line	Chen et al. (2016)
31.	Piperlongumine	Alkaloid	*Piper longum* long pepper	Increased the levels of apoptotic (Bax, Bcl‐2, and caspase‐3) and autophagic proteins (beclin‐1 and LC3B)	In vitro	Human donors of leukemic cells	Xiong et al. (2015)
32.	Indicaxanthin	Alkaloid	Cactus pear *(O. ficus indica)*	Suppressed 7‐ketocholesterol‐induced THP‐1 cell apoptosis	In vitro	THP‐1 cell	Tesoriere et al. (2013)
33.	Cyclopamine	Steroidal alkaloid	*Veratrum californicum*	Inhibited the hedgehog (Hh) signaling pathway	In vitro	HL‐60 cell line	Takahashi et al. (2011)
34.	Tomatidine	Glycoside of the steroid alkaloid	Tomatoes	Inhibited cell growth and induced apoptosis	In vitro	HL‐60 cell line	Huang et al. (2015)
35.	Berberine	Alkaloid	*Berberis vulgaris*	Inhibited SDF‐1‐induced AML cells, and reduced SDF‐1 protein level	In vitro	HL‐60 cell line	Li et al. (2008)
36.	Colchicine	Alkaloid	*Colchicum autumnale*	Complete remission in one patient by blood cell count	In vivo	Peripheral blood	Kneedler (1945)
**Organosulfur compounds**							
37.	Diallyl sulfide (DAS), diallyl disulfide (DADS), dipropyl disulfide, and dimethyl disulfide	Organosulfur compound	Allium vegetables, such as garlic (*Allium sativum*)	More potent HDAC inhibitor, induced histone acetylation and cell growth; docking studies predicted their direct binding to the HDAC as active site and their HDACs inhibitory potential was confirmed by activity assays	In vitro	U937, NB4, HL‐60, and MonoMac‐6 (MM6)	Merhi et al. (2008)
38.	Sulforaphane (SFN)	Isothiocyanate	Cruciferous vegetable	Inhibited cell proliferation and induced apoptosis	In vitro	KG1a and K562 cells	Wang et al. (2018)
**Terpenes and Terpenoids**							
39.	Cantharidin	Terpenoids	Secreted by blister beetles	Decreased hepatic leukemia factor protein levels and ↑apoptosis in the AML cell line Induced p53 and the mitochondrial caspase cascade High toxicity limits its use	In vitro and in vivo	MV4‐11 and AML donors from patients	Dorn et al. (2009)
40.	Celastrol	Pentacyclic triterpenoid	*Tripterygii Wilfordii Radix*	Inhibited the activity of C/EBPβ by disrupting its interaction with the Taz2 domain of p300	In vitro	AML cells	Coulibaly et al. (2018)
41.	Dehydroleucodine	Sesquiterpene lactone	*Genoxys verrucose*	Induced HMOX1 and HSPA1A and downregulated NF‐κB on HL‐60, Kasumi‐1, KG‐1, MOLM‐13, MV4‐11, THP‐1, TUR, and U937	In vitro	8 AML cells and peripheral blood mononuclear cells	Ordóñez et al. (2016)
42.	Helenalin	Sesquiterpene lactone	*Arnica* spp.	Activated mitochondrial‐induced apoptosis	In vitro	HL‐60 cells	Liu et al. (2019)
43.	Friedelin	Triterpenoid	Roots of the Cannabis plant	Induced apoptosis	In vitro	AML‐196 cells	Chang et al. (2020)
44.	Oridonin	Diterpenoid	*Rabdosia rubescen*	Inhibited cell growth	In vitro and in vivo	MV4‐11/DDP, MOLM‐13/DDP cells, and xenografted nude mice	Zhang, Wang, et al. (2017)
45.	Betulinic	Pentacyclic triterpenoid	*Betula pubescens*	Increased aryl hydrocarbon receptor (AHR) expression		HL‐60 and THP‐1	Zhang, Li, et al. (2017)
46.	Ursolic acid	Pentacyclic triterpenoid	Apple skin, marjoram leaves, and rosemary leaves	Induced apoptosis in human leukemia cells in a dose‐ and time‐dependent manner	In vitro	U937, HL‐60, and Jurkat cells	Gao et al. (2012)
47.	Cholestane glycosides	Tetracyclic triterpene	*Chamaelirium luteum*	Cytotoxic to HL‐60 cells, with IC_50_ values of 12.8, 9.8, 15.3, 6.2, and 10.2 lM	In vitro	HL‐60	Yokosuka et al. (2013)
**Other groups (both known and emerging)**							
48.	Silvestrol	Flavagline family	*Aglaia foveolata*	Inhibited cell proliferation, increased apoptosis, inhibited FLT3 and miR‐155 expression	In vitro and in vivo	MV4‐11 leukemia‐engrafted mice	Maior and Dobrotă (2013)
49.	α‐Mangostin	Xanthone	*Garcinia mangostana* (mangosteen tree)	Combination of α‐mangostin, gallic acid, or vitamin C and Dox‐blocked MOLM‐13 cells at G2/M phase, indicating irreversible cell cycle arrest Induced senescence, an antitumorigenic mechanism that inhibits cancer as determined by the p16 expression	In vitro	Relapsed AML cell line MOLM‐13	Osemeke (n.d.)
50.	Caffeic acid phenethyl ester (CAPE)	Hydroxycinnamic acid	Propolis from honey	Targeted the NFκB transcription factor, promoting apoptosis in a wide range of cell lines	In vitro	HL‐60 cells	Budisan et al. (2017)
51.	Curcumin	Curcuminoid	*Curcuma longa* L. (Tumeric)	Induced G1 phase arrest in HL‐60 cells and G2/M phase arrest in K562 cells	In vitro	HL‐60 and K562 cells	Martínez‐Castillo et al. (2018)
52.	Resveratrol (a phytoalexin)	Stilbenoid	Red wine and berries	Inhibited HDACI‐induced RelA/p65 acetylation and NF‐κB activation. Activated caspase‐8. Induced ROS production and S‐phase accumulation	In vitro	U937 and MV‐4‐11	Yaseen et al. (2012)
53.	Emodin	Anthraquinone	Rhubarb, buckthorn, and Japanese knotweed	Decreased cell viability and induced apoptosis	In vitro	KG‐1a and K562) cells	Mahbub et al. (2013)
54.	Glycyrrhizic acid	Saponin	Root of licorice plant (*Glycyrrhiza glabra*)	Inhibited leukemia cell growth and migration by inhibiting AKT/mTOR/STAT3 signaling	In vitro	TF‐1 cells	He et al. (2015)
55.	Hispolon	Polyphenol	*Phellinus igniariu* (Mushroom)	Inhibited HL‐60 leukemia cell line, activated caspase‐8, 9, and 3 by BCL‐2, and upregulated PARP	In vitro	M2: HL‐60; M4: OCI‐ ‐HPNE	Hsiao et al. (2013)
56.	Intermedeol	Eudesmane	*Ligularia fischeri* var.	Antiproliferation activity of intermedeol	In vitro	HL‐60	Jeong et al. (2002)
57.	Miconidine acetate	Hydroquinone	Leaves of *Eugenia hiemalis*	Increased mitochondrial disruption due to ROS production (by 60.0%) Enhanced the proapoptotic Bax protein production	In vitro	K‐562	Maioral et al. (2019)
58.	Arylnaphtalene lignan justicidin B	Lignan	Roots of *Linum leonii*	Apoptosis due to the upregulation of caspase‐3, 8, and 9 enzymes	In vitro	HL‐60	Momekov et al. (2014)
59.	Obovatol	Biphenolic	Bark cortex of *Magnolia officinalis*	Inhibited cell growth, induced apoptosis by regulating the MAPK signaling pathway, and suppressed the expression of MLL target genes by decreasing the phosphorylation of NF‐κB signaling‐associated proteins	In vitro	MM6, THP‐1, and U937 cells	Kim et al. (2014)
60.	20‐Hydroxyecdysone	Ecdysteroid hormone	Bark of *Dacrycarpus imbricatus*	Induced dose‐dependent reduction in the cell proliferation activity of the OCI leukemia cell line tested.	In vitro	OCI leukemia cell line	Thuy et al. (2017)
61.	Combretastatin	Dihydrostilbenoid	*Combretum caffrum*	Remission in human trials	In vivo	Peripheral blood	Uckun et al. (2019)
62.	Indirubin	Indolines	Indigo plant	Inhibited activity of FLT3 and arrested cell cycle of MV4;11 cells at the G1 phase	In vitro	MV4;11 and RS4;11	Han et al. (2010)
63.	Beta‐lapachone	o‐Naphthoquinone	*Tabebuia avellanedae*, and bark of the lapacho tree	Inhibited G0/G1 phases with a decrease in S and G2/M phases and an increase in apoptotic cell populations	In vitro	HL‐60 cells	Planchon et al. (1995)

### 3.2 Discussion

Based on the data obtained from this study (Table 2), we identified a myriad of promising bioactive compounds that have the potential to be used as novel drugs or serve as leads for the discovery and development of new pharmacological agents to target AML cells. Although the majority of the analyzed research consisted of cell models of AML (leukemic cells, in vitro), the effort is commendable and might represent a leading step for future drug discovery studies involving in vivo AML models. From the extracted data, we found that the majority of the compounds belong to one of the following compound classes: polyphenols (mainly flavonoids), alkaloids, organosulfur compounds, and terpenoids.

**FIGURE 3:**
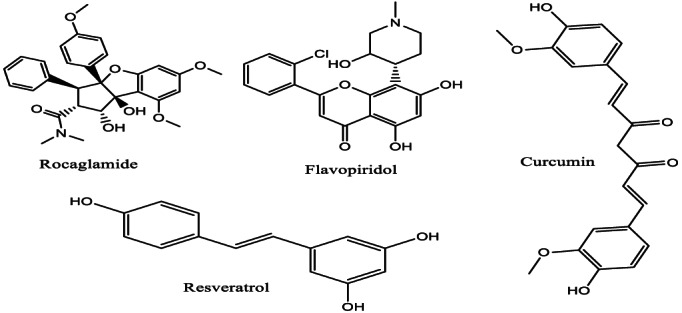
Chemical structures of some flavonoids.

The cytotoxicity of the flavagline family on AML cells, which occurs via several mechanisms, has been studied and established (Menezes et al., 2016; Saraei et al., 2019). Epigallocatechin‐3‐gallate has been reported to be effective against AML. The significant reduction in the xenograft capability of rocaglamide‐treated AML cells shows that rocaglamide targets leukemia stem cells (LSCs) and has an insignificant influence on hematopoietic stem cells (HSCs). Silvestrol and rocaglamide downregulate Myc proteins, disrupt the integrity of the mitochondria, reduce antiapoptotic proteins, and inhibit translation, all of which are essential for LSCs self‐renewal (Callahan et al., 2014; Saraei et al., 2019). Rocaglamide (Figure 3) inhibits the eukaryotic initiation factor 4E phosphorylation by preventing Erk (extracellular signal‐regulated kinase; Iwasaki et al., 2019). Silvestrol prevents the overexpression of FLT3 and reduces miR‐155 levels, one of the regulators of FLT3 internal tandem duplication (ITD)‐positive AML cells (Alachkar et al., 2013).

A flavopiridol (Figure 3) known as alvocidib effectively inhibits CDK9 (cyclin‐dependent kinase 9) and causes cyclin D1, Myc, and Mcl‐1 transcription repression. Several clinical trials involving alvocidib indicate the potential effectiveness in patients with newly diagnosed and refractory/relapsed (R/R) AML (Lee & Zeidner, 2019). Newly diagnosed AML patients who were treated using the 7 + 3 induction chemotherapy cycle were recruited for the clinical trial and benefited from gene expression profile analyses. Refractory patients were distributed into three groups based on the different profiles of gene expressions. In patients with refractory AML, leukemic cells were sensitive to alvocidib, indicating that this flavopiridol (alvocidib) is a promising candidate for relapsed/refractory AML patients (Horibata et al., 2019).

Curcumin (Figure 3) is a phenol with cytotoxic effects on LSCs and leukemia cells. Following treatment with curcumin, the mRNA levels of the signal transducer and activator of transcription 3 (STAT3) and KG1 cells' BCL‐XL decreased (Mohammadi Kian et al., 2020). Curcumin significantly decreased the population of ALDH+ cells in the THP‐1 cell line model. In addition, curcumin inhibited cell proliferation via the inhibition of the Notch and Hedgehog pathways (Li, Domina, et al., 2018). Curcumin, in combination with nanoliposome carriers, showed a high cell uptake and satisfactory targeting capability. The curcumin + nanoliposome carriers’ combination significantly improved the survival of mice with AML. The c‐Myc inhibition by curcumin in combination with nanoliposome carriers also led to the downregulation of the expression of DNMT1, resulting in inadequate gene methylation and leading thus to the re‐expression of downstream tumor suppressor genes, for example, miR‐223. Curcumin in combination with nanoliposome carriers also resulted in caspase‐dependent apoptosis by hindering the MAPK and Akt/mTOR pathways (Sun et al., 2017).

Resveratrol (Figure 3) is a polyphenol that occurs in several plants, including peanuts(Awuchi & Okpala, 2022). It inhibits the survival of AML cells via activation of Fas‐mediated apoptosis and caspase signaling. The STAT3 signaling inhibition and the downregulation of the expression of BCL‐2 (B‐cell lymphoma 2) also partake in the resveratrol‐induced apoptosis (Huang, Li, et al., 2019). Resveratrol could reverse the drug resistance of AML cells through PI3K/Akt/Nrf2 signal regulation (Li et al., 2019). Several studies have indicated that resveratrol inhibits cancer stem cells. It has been shown to inhibit the growth of KG1 cells. Resveratrol stimulates the upregulation of the ligands of the “natural‐killer group 2, member D" (NKG2D) for ULBP1/2/3. Resveratrol reduces the DcR1 (decoy receptor 1) expression and encourages the DR4 (death receptor 4) upregulation. All these actions enhance the KG1 cells' sensitivity to the cytolysis, mediated by the CIKs (cytokine‐induced killer cells; Hu et al., 2012).

The metabolites of flavonoids enhance the susceptibility of resistant malignant cells to chemotherapy drugs and, consequently, have been acknowledged as effective options in the management of AML. Many studies have shown that flavonoids can overcome the resistance of death ligand agonists in various cellular models of leukemia (Russo et al., 2014; Nwozo et al., 2023). Quercetin, a popular flavonoid, has been reported to enhance the susceptibility of chronic lymphocytic leukemia (CLL) cells to anti‐CD95 molecules and to the recombinant “TNF‐related apoptosis‐inducing ligand" (TRAIL), as well as to fludarabine, which is a drug widely used in the treatment of CLL (Russo et al., 2010). Additionally, TRAIL and 4′‐bromoflavonol combined treatments enhance the leukemic cells' susceptibility to TRAIL (Burmistrova et al., 2014). Studies also show that this flavonoid has pro‐apoptotic properties and, as a result, it increases the anticancer potency of chemotherapeutic drugs in multidrug‐resistant cells, for example, the P388 leukemia cell line. It appears that flavonoids have the potential to emerge as sensitizer/chemopreventive compounds and compliment the efficacy of chemotherapeutic agents in multidrug‐resistant leukemia cells (Gatouillat et al., 2015). Other flavonoids that have demonstrated antileukemic functions are chrysin in CLL, myricetin and baicalein in chronic myeloid leukemia (CML), wogonoside in T‐cell acute lymphoblastic leukemia (T‐ALL), and catechin and alvocidib in AML (Mukhopadhyay et al., 2017; Romanouskaya & Grinev, 2009; Salimi et al., 2017).

**FIGURE 4:**
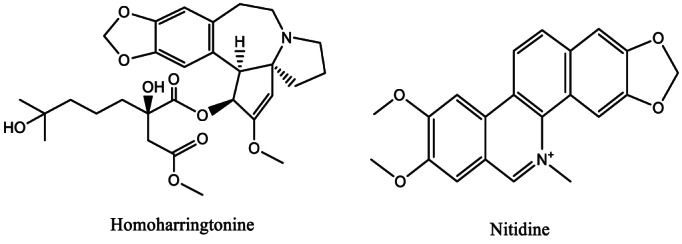
Chemical structures of homoharringtonine and nitidine.

Alkaloids are complex and large group of cyclic compounds that contain at least one atom of nitrogen (Figure 4). They are found in almost all plant groups, fungi, and algae species, with over 12,000 of them isolated so far. Wei et al. (2020) reported that alkaloid‐based regimens show undisputed efficacy in AML treatment. Feldman et al. (1996) emphasized that the conventional cephalotaxine alkaloid named homoharringtonine (Figure 4) is beneficial in reducing the complications of myelodysplastic syndromes which can evolve into AML. Bacher et al. (2006) reported that oxindole alkaloids from *Uncaria tomentosa* are able to induce apoptosis in G0/G1‐arrested, proliferating, and B‐cell lymphoma 2 (bcl‐2)‐expressing AML cells. Another study by Ye et al. (2016) showed that the novel amaryllidaceae alkaloid named N‐ amaryllidaceae chloride (NMHC) extracted from *Zaphyranthes candida* possesses an enhanced ability to inhibit AML in vivo. The authors hypothesized that this alkaloid caused an aberrant activation of the Notch signaling pathway by docking into the hydrophobic cavity to promote the NOTCH1 proteolytic cleavage via the NOTCH1‐negative regulatory region (Ye et al., 2016). On the same hand, Huang, Pan, et al. (2019) reported that a novel Bruton's tyrosine kinase (BTK) inhibitor named abivertinib (another alkaloid) possesses antileukemia properties and synergistic effects against AML cells along with homoharringtonine. In a different study by Li, Yan, et al. (2018), it was stated that a natural alkaloid named nitidine chloride possesses an enhanced ability to arrest the cell cycle and induce apoptosis in AML cells. The study revealed that nitidine chloride possesses a dose‐ and time‐dependent growth inhibition activity against AML cells after 48 h of treatment by downregulating the cyclins B1, BCL‐2, and cyclin‐dependent kinase‐1 (CDK‐1; Li, Yan, et al., 2018). Nitidine also upregulates the p27‐ and bcl‐2‐like protein 4 (Bax) while inactivating the poly‐(ADP ribose) polymerase (PARP; Li, Yan, et al., 2018). It also activates caspase‐3 as well as inhibits the phosphorylation of protein kinase B (AKT) and extracellular signal‐regulated kinase (ERK; Li, Yan, et al., 2018). It is evident from all these studies that alkaloids exhibit an enhanced potential to inhibit the growth and development of AML cells.

Organosulfur compounds (OSCs) are sulfur‐containing organic compounds (Figure 4) found abundantly in nature—plants and animals. Sulfur‐containing compounds are essential for human life, for example, amino acids like cysteine and methionine contain sulfur in their chemical structure. Organosulfur compounds extracted from allium species (chives, garlic, leek, onion, scallions, and shallots) showed antiproliferative and apoptotic effects in human cell lines (U97, NB4, HL‐60, and MonoMac‐6; Auger et al., 2008). OSCs from garlic have been the most studied in the management of AML. Garlic OSCs can be classified as thiosulfinates and sulfides. Alliin, a (+)S‐allyl‐L‐cysteine sulfoxide, is present in the intact garlic clove and is converted into the corresponding thiosulfinate (Allicin) by the enzyme allinase when the garlic clove is cut, crushed, chopped, or chewed. Allicin is unstable, it rapidly degrades and rearranges to stable derivatives. The anti‐AML activity is related to the disulfide functional group present in these compounds (Kaschula et al., 2011). Ajoene in combination with conventional chemotherapeutic agents has shown promising effects in AML therapy (Hassan, 2004). Chemically, ajoene is a Z,E 4,5,9‐trithiadodeca‐1,6,11‐triene‐9‐oxide and exhibits geometric isomerism (E‐ and Z‐isomers). Block et al. (1984, 1986) demonstrated the mechanism of rearrangement of allicin to ajoene. Ajoene is chemically more stable than allicin and contains two functional groups (sulfoxide and disulfide). Chaetocin is a specific inhibitor of histone lysine N‐methyltransferase SUV39H1 enzyme which interacts with the proto‐oncogenes involved in AML development. Chaetocin is a fungal mycotoxin derived from Chaetomium fungal species (Lai et al., 2015).

**FIGURE 5:**
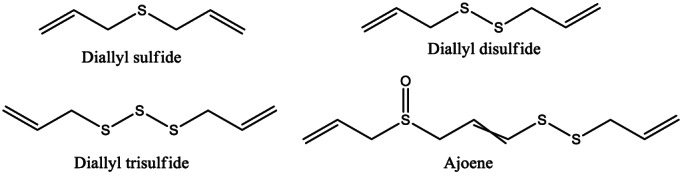
Chemical structures of some organosulfur compounds.

Organosulfur compounds such as diallyl sulfide (DAS), diallyl disulfide (DADS), diallyl trisulfide (DATS), and thiosulfonate, deoxysulfone, and ajoene (Figure 5) exhibit antileukemic effects via several mechanisms, namely by inducing cell cycle arrest and apoptosis in cellular models of leukemia.

In terms of the apoptotic effects of OSCs, studies have suggested that OSCs can induce apoptosis in leukemic cells. The mechanism of OSCs‐induced apoptosis involves many features. The responsible mechanism for apoptosis mainly involves the release of ROS as a result of cell damage and moderate elevation of exogenous oxidants such as H_2_O_2_ (Wu et al., 2005). DADS promoted the apoptosis of HL‐60 cells via an increased expression of the GTPase Ras‐related C3 botulinum toxin substrate 2 (Rac2). Furthermore, research has shown that Rac2, NADPH oxidase, and ROS have important roles in the apoptosis induced by DADS in HL‐60 cells (Yi, Ji, Lin, et al., 2010; Yi, Ji, Tan, et al., 2010). DADS‐induced apoptosis in human leukemia HL‐60 cells is triggered by caspase‐3 activation, poly(adenosine diphosphate‐ribose) polymerase (PARP) degradation, and DNA fragmentation (Kwon et al., 2002). Moreover, the DADS‐mediated apoptosis in HL‐60 cells also involves the activation of JNK via ROS generation (Novotny‐Diermayr et al., 2012). Other evidence suggests that ERK signaling pathway inhibition and activation of the p38 signaling pathway in DADS leads to apoptosis (Tan et al., 2008). The induction of apoptosis in human leukemia K562 cells by DADS involves the activation of the Fas/FasL pathway which is associated with an increased expression of the Fas gene and caspase‐8 and a decreased expression of FasL and Bag‐1 genes (Lin et al., 2007; Xiao et al., 2011; Yin & Peng, 2011). DATS inhibited the growth of U937 leukemia cells by apoptosis induction in a concentration‐ and time‐dependent manner. DATS‐induced apoptosis was associated with the downregulation of the protein levels of Bcl‐2, XIAP, and cIAP‐1, the cleavage of Bid proteins, the activation of caspases, and the collapse of the mitochondrial membrane potential. Furthermore, the data showed that DATS increased the generation of intracellular ROS, which was attenuated by pretreatment with N‐acetyl‐L‐cysteine (NAC), an antioxidant that acts as a ROS scavenger (Agassi et al., 2020; Choi & Park, 2012). Furthermore, NAC administration resulted in a major inhibition of the DATS‐induced apoptosis by inhibiting caspase activation. Apoptosis induction in HL‐60 cells treated with moderate doses of DADS may be associated with the expression of DJ‐1 (also known as Parkinsonism‐associated deglycase‐7, PARK‐7) in the mitochondria (Li, Tang, et al., 2016). Moreover, the aforementioned effect is extrapolated to the activation of caspase‐9, an initiator caspase of the mitochondrial‐mediated intrinsic pathway, and caspase‐3, accompanied by the proteolytic degradation of poly(ADP‐ribose)‐polymerase. In one of the investigations, the DADS lead to apoptosis and autophagy via mTOR (mammalian target of rapamycin) activation in K562 and NB4 myeloid leukemia cell lines (Suangtamai & Tanyong, 2016). In several cell lines of leukemia, for example, U937, K562, and Jurkat, since they lack a wild‐type p53, NF‐κB‐mediated pathways might play another putative role in the apoptosis mediated by the DADS treatment. DADS induced reversible G2/M arrest via an increased nuclear translocation of NF‐κB and its specific binding to the p21 promoter (Tiong & Wei, 2019).

In animal models, terpenoids have been shown to possess chemopreventive properties as well as in the treatment of cancer (Haider et al., 2014; Tiong & Wei, 2019). The plant‐derived molecule, helenalin (sesquiterpene lactone) has been investigated against doxorubicin‐resistant AML HL‐60 cells. It was found to induce apoptosis, cell inhibition, cell migration, and Akt/PI3K/mTOR signaling pathway inhibition (Liu et al., 2019). Helenalin strongly inhibited HL‐60 cell growth and displayed an IC_50_ of 23.5 μM. It was noted that helenalin's anticancer effects are due to the activation of mitochondrial‐induced apoptosis which has also been associated with an increase in Bax expression and a decrease in Bcl‐2 expression. Helenalin also triggered the loss of MMP in HL‐60 doxorubicin‐resistant cells and also blocked their migratory and invasive properties via signaling modulation (Babaei et al., 2018; Drogosz & Janecka, 2019).

The development of human leukemia was substantially suppressed by friedelin by means of apoptosis induction. With no effect on normal cells, friedelin blocked the proliferation of human AML‐196 cells. The review of its underlying mechanisms found that, in AML‐196 cells, friedelin mediated apoptosis. Apoptosis induced by friedelin was associated with the upregulation of the cleaved caspase‐3, 8, and 9 as well as of the cleaved PARP. The levels of Bax protein were elevated and the levels of Bcl‐2 were lowered (Asati et al., 2016; Chang et al., 2020). In addition, friedelin also blocked the MEK/ERK and PI3K/AKT signaling in a dose‐dependent fashion (Zhou et al., 2019).

Matrix metalloproteinases (MMPs) are involved in the processes of invasion and metastasis in human malignancies, and the expression levels of MMP‐2 and MMP‐9 have been shown to decrease in MV4‐11/DDP cells treated with high oridonin concentrations. Oridonin may have the capacity to suppress human AML cell invasion and metastasis and is likely to be effective in inhibiting the development of drug‐resistant AML cells. Combination treatment with oridonin and cisplatin has become a useful way of treating human AML cells resistant to cisplatin. Both compounds exerted synergistic antitumor effects, and cisplatin tolerance in human AML cells was essentially reversed. Meanwhile, under these laboratory conditions, opioid toxicity had to be minimized (Zhang, Wang, et al., 2017).

Besides terrestrial organisms, marine organisms are also valuable sources of anticancer agents such as peptides, alkaloids, polyketides, phloroglucinols, polyphenols, sulfated polysaccharides, sterols, carotenoids, and polysaccharide derivatives obtained from chitin, chitosan, and chitooligosaccharides, etc. (Chakraborty et al., 2009; Simmons et al., 2005; Sithranga Boopathy & Kathiresan, 2010; Wang, Sorolla, et al., 2020). Polyphenols (ecol, diecol, phloroglucinal, and phlorofucofuroecol A), alkaloids (brugine and benzoxazolinone), antibiotics (daunorubicin), and polysaccharides (chondroitin‐6‐sulphate, chondroitin‐4‐sulphate, heparin, and fucoidan) are some notable biologically potent and predominant classes of anticancer natural products derived from marine organisms (Table 3; Simmons et al., 2005; Sithranga Boopathy & Kathiresan, 2010). To sum up, our systematic review identified potential molecules with anti‐AML properties which could emerge as leads for novel pharmacological agents targeting AML cells and improving patient care worldwide.

**TABLE 3:** Marine organisms‐derived bioactive compounds effective against AML (Alves et al., 2018; Folmer et al., 2010; Schwartsmann et al., 2001; Sithranga Boopathy & Kathiresan, 2010; Wali et al., 2019; Wang, Sorolla, et al., 2020).

Marine organism	Species	Bioactive compound	Type
Actinomycetes	*Streptomyces peucetius*	Daunorubicin	Anthracycline antibiotic
Bryozoan	*Bugula neritina*	Bryostatin 1	Macrocyclic lactone
Macroalgae	*Hydroclathrus clathratus*	H3‐a1	Sulfated polysaccharide
Mollusk	*Jorunna funebris*	Jorumycin	Isoquinoline alkaloid
Sponge	*Cryptotethya crypta*	Ara‐C (Cytarabine)	Pyrimidine nucleoside
Sponge	*Halichondria okadai*	Halichondrin B	Polyether macrolide
Sponge	*Pseudaxinyssa cantharella*	Girodazole	Imidazole alkaloid
Tunicate	*Didemnum cuculiferum*	Vitilevuamide	Bicyclic peptide
Tunicate	*Eudistoma olivaceum*	Eudistomin	Amino acid‐derived alkaloid
Tunicate	*Eudistoma toealensis*	Staurosporine	Indolocarbazole alkaloid

## 4 CONCLUSION

Acute myeloid leukemia is one of the most common kinds of leukemia, resulting in significant human mortality in both children and adults. It is a type of hematological malignancy characterized by dysregulation of cell differentiation, proliferation, and death induced by epigenetic and genetic changes in hematopoietic progenitor or stem cells. Despite the development of innovative treatments in recent years, such as modifications in conventional chemotherapies, hypomethylating medicines, and molecular targeted medications, resistance and relapse remain the key clinical concerns of AML care. As a result, there is an urgent need for ongoing research on better alternatives. This study has identified various promising bioactive compounds, many of which pertain to the class of flavonoids and polyphenols. Other important groups are alkaloids, organosulfur compounds and terpenoids. All of the identified compounds showed promising effects on AML cells.

## CONFLICT OF INTEREST STATEMENT

The authors declare no conflict of interest.

## FUNDING INFORMATION

None.

## Data Availability

Additional data will be made available on request.
